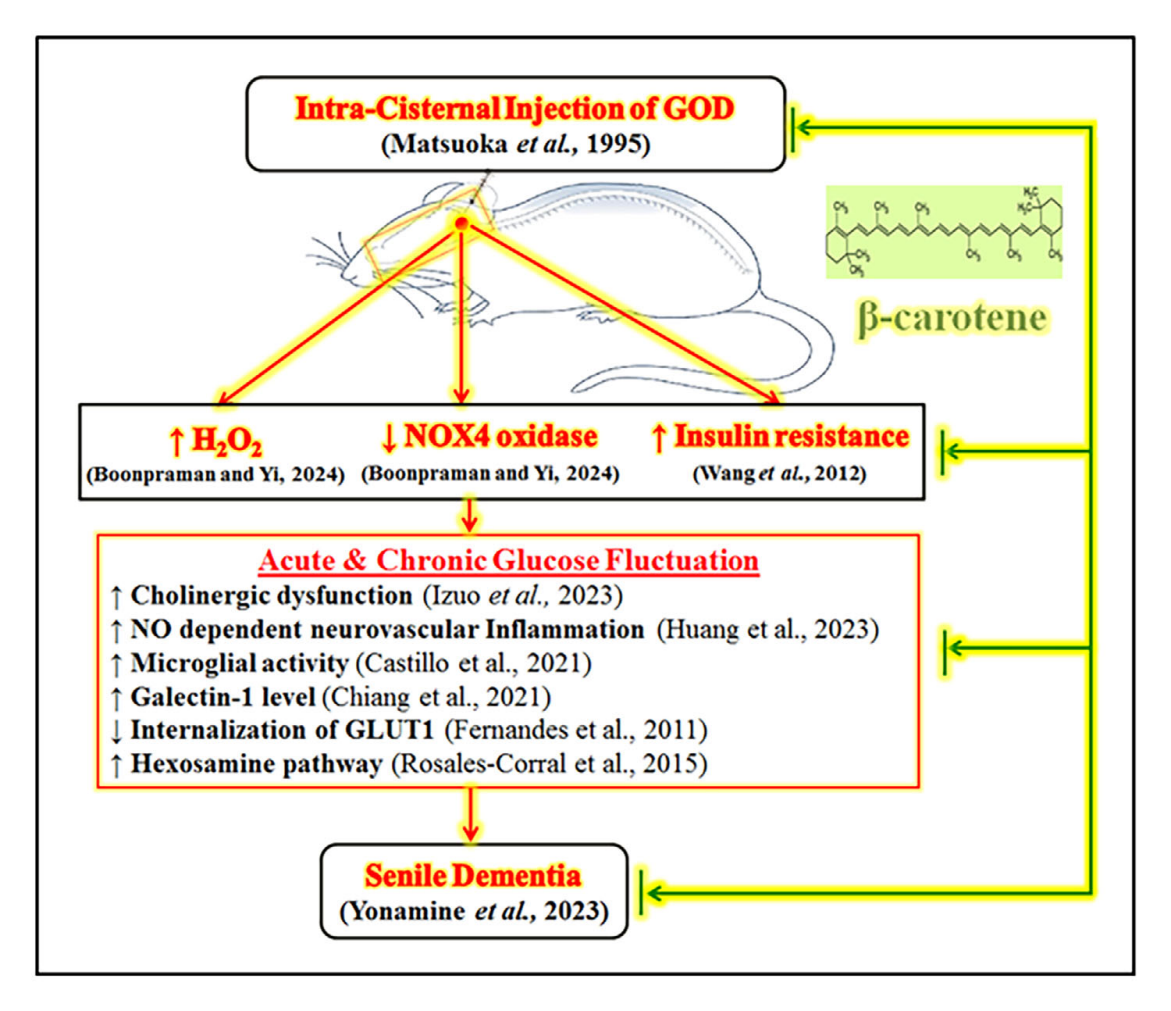# Therapeutic potential of beta‐carotene on intracisternal injection of glucose oxidase‐induced senile dementia in diabetic rats

**DOI:** 10.1002/alz.088448

**Published:** 2025-01-09

**Authors:** Arunachalam Muthuraman, Khian Giap Lim

**Affiliations:** ^1^ AMIST University, Semeling, Kedah Malaysia; ^2^ AIMST University, Bedong, Kedah Malaysia

## Abstract

**Background:**

Senile dementia (SD) is a deteriorative organic brain disorder and it comprises Alzheimer’s disease (AD) as a major variant. SD is shown impairment of mental capacities whereas AD is degeneration of neurons. According to World Health Organization (WHO) report; more than 55 million peoples have dementia and it is raising 10 million new cases every year. Further, it is expected to rise to 78 million in 2030 and triple by 2050. The glucose oxidase (E.C. 1.1.3.4; GOD) is an oxidoreductase enzyme and it oxidizes the β‐d‐glucose to d‐glucono‐δ‐lactone and hydrogen peroxide (H_2_O_2_). In the neuronal system, GOD & H_2_O_2_ cause rapid fluctuations of glucose levels, insulin resistance, oxidative stress along with microglial activation and neurodegenerations. Besides, only a few medications were approved for the symptomatic relief of SD *i.e.*, donepezil, galantamine, rivastigmine, and memantine. Moreover, the most efficient and potential agents are not discovered yet. Hence, the present study was designed to investigate the beta‐carotene (BC) actions against the GOD‐associated SD in diabetic rats.

**Method:**

Male Sprague Dawley rats were used for the induction of diabetes by intraperitoneal injection of nicotinamide (50 mg/kg and streptozotocin 50 mg/kg), and SD was induced by intracisternal administration of GOD (50 U/5 µl at 1 µL/minute). The BC (50 and 100 mg/kg; *p.o*.) and donepezil (1 mg/kg; *p.o*.) were administered for 15 consecutive days. The cognitive function was assessed by the Morris water maze test and biomarkers i.e., blood glucose & insulin; serum nitric oxide (NO); tissue acetylcholinesterase (AChE), galectin‐1 (G1), NADPH oxidase (NOX4) activity & glucose transporter 1 (GLUT1) levels were evaluated.

**Result:**

GOD potentially changes the neurovascular unit in the brain which leads to a rise the insulin resistance (IR), NO, G1, & GLUT1 levels; and decreases the NOX4 activity. The GOD causes the potential cognitive dysfunctions. However, the treatment of BC attenuated the GOD‐associated cognitive dysfunction and biomarker changes.

**Conclusion:**

The present results revealed that BC possesses the ameliorative potential against GOD‐induced neurotoxicity and SD due to its anti‐oxidative, anti‐cholinesterase, reduction of IR, prevention of microglial activation, and enhancement of the glucose update actions.